# Correlation of* A2bAR* and* KLF4/KLF15* with Obesity-Dyslipidemia Induced Inflammation in Uygur Population

**DOI:** 10.1155/2016/7015620

**Published:** 2016-04-21

**Authors:** Cuizhe Wang, Xiaodan Ha, Wei Li, Peng Xu, Yajuan Gu, Tingting Wang, Yan Wang, Jianxin Xie, Jun Zhang

**Affiliations:** ^1^Shihezi University School of Medicine, Shihezi, Xinjiang 832000, China; ^2^The First Affiliated Hospital Clinical Laboratory, Shihezi University School of Medicine, Shihezi, Xinjiang 832000, China; ^3^Endocrinology Department, Xinjiang Uygur Autonomous Region People's Hospital, Urumqi, Xinjiang 830001, China

## Abstract

In this paper, the researchers collected visceral adipose tissue from the Uygur population, which were divided into two groups: the normal control group (NC, *n* = 50, 18.0 kg/m^2^ ≤ BMI ≤ 23.9 kg/m^2^) and the obese group (OB, *n* = 45, BMI ≥ 28 kg/m^2^), and then use real-time PCR to detect the mRNA expression level of key genes involved in inflammation signaling pathway. The findings suggest that, in obese status, the lower expression level of* A2bAR*,* KLF4*, and* KLF15* of visceral adipose tissue may correlate with obese-dyslipidemia induced inflammation in Uygur population.

## 1. Introduction

Obesity, particularly abdominal obesity, has become worldwide health problem, which is closely related to the increased lipolysis of adipocyte and blood lipid level [1]. Importantly, lipid metabolism disorder is closely related to chronic inflammation induced by obesity [2]. The Uygur and Kazak are two of the main minorities of Xinjiang Region, the former of which is more likely to suffer obesity and type 2 diabetes mellitus (T2DM) [3]. Our group has previously reported that at the same body mass index (BMI) level both male and female Uygur subjects had significantly greater value of WHR and visceral fat content, compared with Kazak subjects [3]. It remains unclear why Uygur population is more susceptible to obesity and T2DM.

Kruppel-like factors (KLFs), as a transcription factor family, are composed of 17 members with zinc finger structure, widely involved in cell proliferation, differentiation, and embryonic developmental regulation [4]. KLF4 has anti-inflammation effects and can promote fatty acid *β*-oxidation [5, 6]. KLF15, like KLF4, is a newly discovered transcription factor that plays an important role in glucose homeostasis and lipid accumulation in cells [7]. The A2b adenosine receptor (A2bAR) is expressed on the cell membrane and involved in lipid metabolism and inflammation [8, 9]. Visceral adipose tissue, as one of the principal locations of the systemic inflammation response, plays an important role in the regulation of body energy metabolism [10]. In the process of inflammation induced by abdominal obesity, whether KLF4 and KLF15 play an important role and whether the A2bAR correlates with KLF4 and KLF15 are not found.

Thus, our study intends to evaluate the mRNA expression level of* A2bAR*,* KLF4/KLF15,* and key inflammation signaling pathway genes in visceral adipose tissue from Uygur population to investigate the correlation of* A2bAR *and* KLF4/KLF15* with obesity-dyslipidemia induced inflammation in Uygur population.

## 2. Materials and Methods

### 2.1. Subjects

We enrolled 172 Uygur subjects between the ages of 20 and 90 years between January and December 2014 from People's Hospital of Xinjiang Uygur Autonomous Region for physical examination and evaluation of dyslipidemia. We collected visceral adipose tissue from 95 Uygur subjects and analyzed mRNA expression level of key genes related to inflammation. Those subjects were divided into two groups: the normal control group (NC, *n* = 50, 18.0 kg/m^2^ ≤ BMI ≤ 23.9 kg/m^2^) and the obese group (OB, *n* = 45, BMI ≥ 28 kg/m^2^). Exclusion criteria included type 1 diabetes (T1DM): various pathogenic factors that resulted in the lacking source of insulin and the fasting insulin that is lower than 5 *μ* IU/mL; tumors: the patients who were diagnosed with all kinds of tumors by the doctor; acute inflammation: the patients with the symptoms including sudden onset, short duration, and the granulocyte infiltration that were diagnosed with acute inflammation; kidney disease: the patients with massive proteinuria, hypoproteinemia, and hyperproteinemia; and, what is more, the patients who recently use drugs to interfere with glucose and lipid metabolism.

### 2.2. Anthropometric and Clinical Parameters

We measured the following clinical parameters using standard procedures: height, weight, body mass index (BMI), waist circumference (WC), hip circumference (HC), waist-to-hip ratio (WHR), systolic blood pressure (SBP), and diastolic blood pressure (DBP). BMI was calculated by dividing weight (in kilograms) by height (in meters) squared. WC and HC were measured using a flexible tape with tension calipers at the extremity (Gulick-Creative Health Product, Inc., Plymouth, MI), midway between the xiphoid and umbilicus during the midexpiratory phase and at the maximum circumference in the hip area, respectively. WHR was calculated by dividing WC by HC.

### 2.3. Measurement of Biochemical Indexes

The fasting plasma glucose was detected using the glucose oxidase-peroxidase method [11]. Total cholesterol (TC), triglyceride (TG), high density lipoprotein cholesterol (HDL-C), and low density lipoprotein cholesterol (LDL-C) were detected using a standardized automatic biochemistry analyzer (Japan, Olympus AU2700). Low HDL-C, high TC, high LDL-C, and high TG were defined as HDL-C < 1.03 mmol/L, TC > 5.17 mmol/L, LDL-C > 2.59 mmol/L, and TG > 1.70 mmol/L, respectively. The subjects who have any of the above indexes of abnormal blood lipid level were defined as dyslipidemia [12].

### 2.4. Tissue Samples

On the day of abdominal surgery, we take the visceral adipose tissue whose size is about 3 cm × 3 cm and avoid the burning of sampling process. After the finish of the sampling process, we repeatedly wash the sample three times with PBS buffer solution and then take the visceral adipose tissue into the cryopreserved tubes, tagging name, gender, medical record number, and group. Snap-freezing in liquid nitrogen until RNA extraction was performed. Total RNA was extracted from the tissue within a week and then stored at −80°C.

### 2.5. RNA Isolation and Real-Time PCR

Total RNA was isolated from visceral adipose tissue using TRIZOL reagent (cat. # 15596-026, Life Technologies, Carlsbad, CA, USA) and purified using an RNeasy Mini Kit (cat. # 74106, QIAGEN, GmbH, Germany). RNA purity was evaluated using an Agilent 2100 Bioanalyzer (Agilent Technologies, Santa Clara, CA, USA). Reverse transcription was performed as follows: 25°C for 5 min, 42°C for 60 min, and 70°C for 15 min. Real-time PCR was performed using SYBR Premix Ex Taq (Takara) on a 7500 Real-Time PCR System (Applied Biosystems, Foster City, CA). Primer sequences were listed in Table 1, using GAPDH as an internal control. One microliter (1 *μ*L) of each RT reaction product was amplified in a 20 *μ*L PCR reaction using an ABI Prism 7500 Sequence Detection System (Applied Biosystems). The PCR protocol was performed as follows: 95°C for 30 s and 40 cycles consisting of 5 s at 95°C and 34 s at 60°C. Dissociation curves were analyzed using the Dissociation Curve 1.0 Software (Applied Biosystems) to detect and eliminate possible primer-dimer artifacts. All reactions were performed in triplicate. The relative amounts of target gene transcripts were calculated using the comparative cycle-time method.

### 2.6. Subject's Consent and Ethics Statement

All subjects provided informed and voluntary consent prior to enrollment in this study. This consent included understanding that clinical information and biological samples would be used for research. The consent form and ethical approval were provided by the Medical Ethics Committee at First Affiliated Hospital, Shihezi University School of Medicine (reference number 2014LL22).

### 2.7. Statistical Analysis

SPSS statistical package (version 13.0, SPSS Inc., Chicago, IL, USA) was used for data analysis. Clinical characteristics and biochemical data were expressed as mean ± SD; mRNA expression levels were expressed as mean ± SEM.* t*-test was used for the comparison between different groups. The correlation analysis was tested by Pearson method, and *P* value < 0.05 was defined as statistical significance.

### 2.8. Quality Control of Laboratory Testing

This study implemented strict quality control methods for the collection of subjects' general information and visceral adipose tissue. A team of researchers designed questionnaires and two independent operators performed molecular biology techniques. Attempts were made to choose subjects from different group who were well matched in terms of age and gender.

## 3. Results 

### 3.1. The Detection Rate of Dyslipidemia in Uygur Population

The detection rate of total dyslipidemia was 61.05%, with increasing of age; the detection rate of high blood TC was 14.53% and similarly demonstrated an increasing trend with age; the detection rate of high blood TG was 22.67%, of which detection rate decreased with the age in men but increased in women; the detection rate of low HDL-C was 42.44%; the detection rate of high LDL-C was 3.49%. The above indexes were higher than those observed in Han and Kazak population except the detection rate of high LDL-C (Tables S1–S6 in Supplementary Material available online at http://dx.doi.org/10.1155/2016/7015620).

### 3.2. Clinical Characteristics of Uygur Subjects in the NC and OB Group

We selected 95 Uygur subjects and divided them into two groups: NC group (*n* = 50) and OB group (*n* = 45). The clinical characteristics were shown in Table 2. The SBP, DBP, and TC in the OB group were higher than in the NC group but were not statistically significant. The weight, WC, HC, WHR, BMI, TG, and TC of individuals in the OB group were significantly higher than those in the NC group (*P* < 0.05).

### 3.3. mRNA Expression of Key Genes in Inflammation Signaling Pathway of Visceral Adipose Tissue

**Figure 1:**
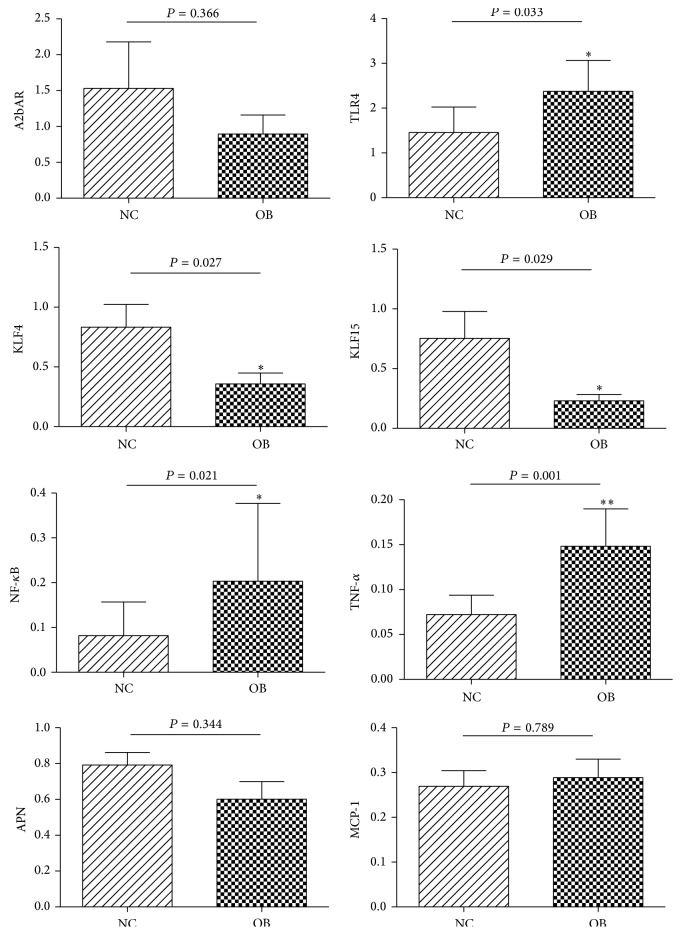
The mRNA expression level of critical gene in inflammation signaling pathways. *t*-test, ^*∗*^ *P* < 0.05, ^*∗∗*^ *P* < 0.01. The difference between the two groups has statistical significance.

The chip analysis revealed significantly differential higher expression of* KLF4* and* KLF15* in normal weight subjects of Uygur population compared to obese subjects, while there is no difference in the Kazak or Han population, respectively (Figure S1, Table S7). Then, we evaluated the key genes mRNA expression level of visceral adipose tissue from NC and OB group in Uygur population. The results were shown in Figure 1. Compared with the NC group, the mRNA expression level of* MCP-1* was slightly higher in the OB group; however this difference was not statistically significant. The levels of* TLR4*,* NF-κB,* and* TNF-α*were significantly higher in the OB group as compared to the NC group (*P* < 0.05), while the expression levels of* A2bAR* and* APN* were lower in the OB group, and the levels of* KLF4* and* KLF15* were significantly lower in the OB group (*P* < 0.05).

### 3.4. The Correlation of* KLF4*/*KLF15* and Dyslipidemia Indexes in OB Group

**Figure 2:**
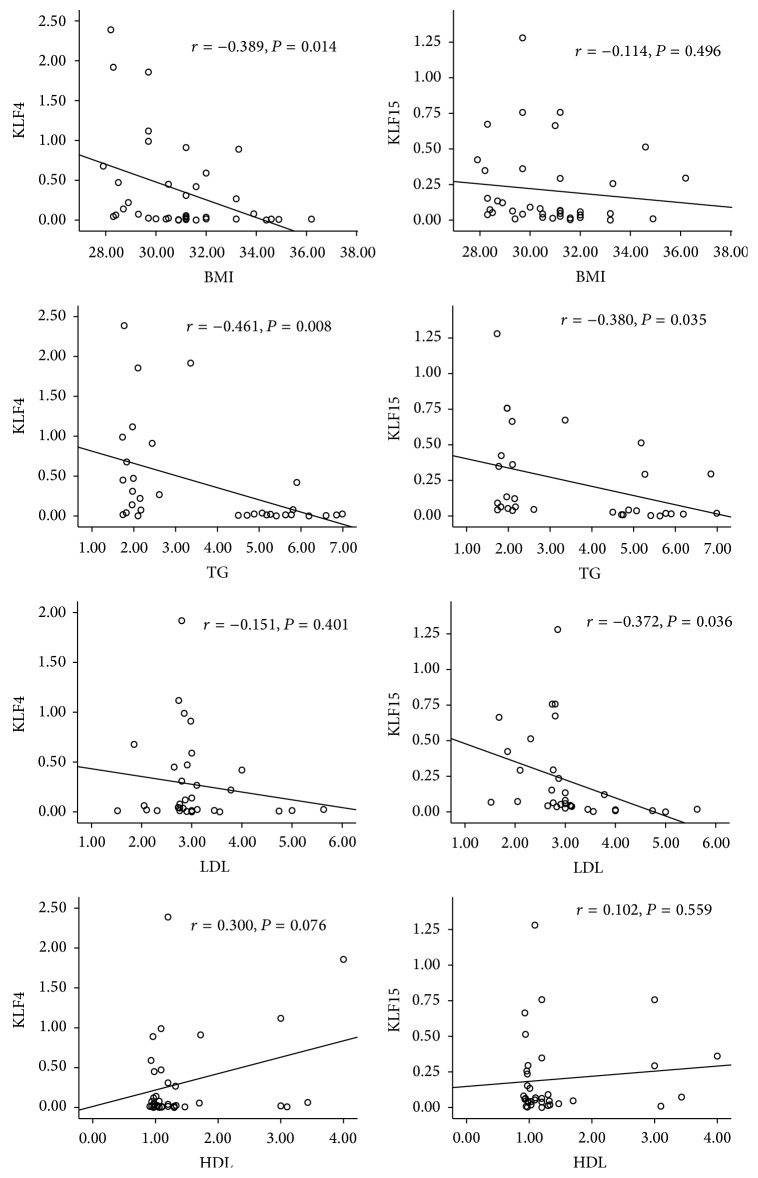
The correlation of the* KLF4*,* KLF15,* and dyslipidemia indexes. Pearson analysis, *P* < 0.05. The correlation between the two groups has statistical significance.

The results were shown in Figure 2. In OB group, the mRNA expression level of* KLF4* was significantly negatively correlated with BMI, TG (*P* < 0.05), and negatively correlated with LDL while* KLF4* was positively correlated with HDL. The mRNA expression level of* KLF15* was significantly negatively correlated with TG and LDL (*P* < 0.05) and negatively correlated with BMI, while* KLF15* was positively correlated with HDL.

### 3.5. The Correlation of* KLF4*/*KLF15* and Key Genes of Inflammation Signal Pathway in OB Group

**Figure 3:**
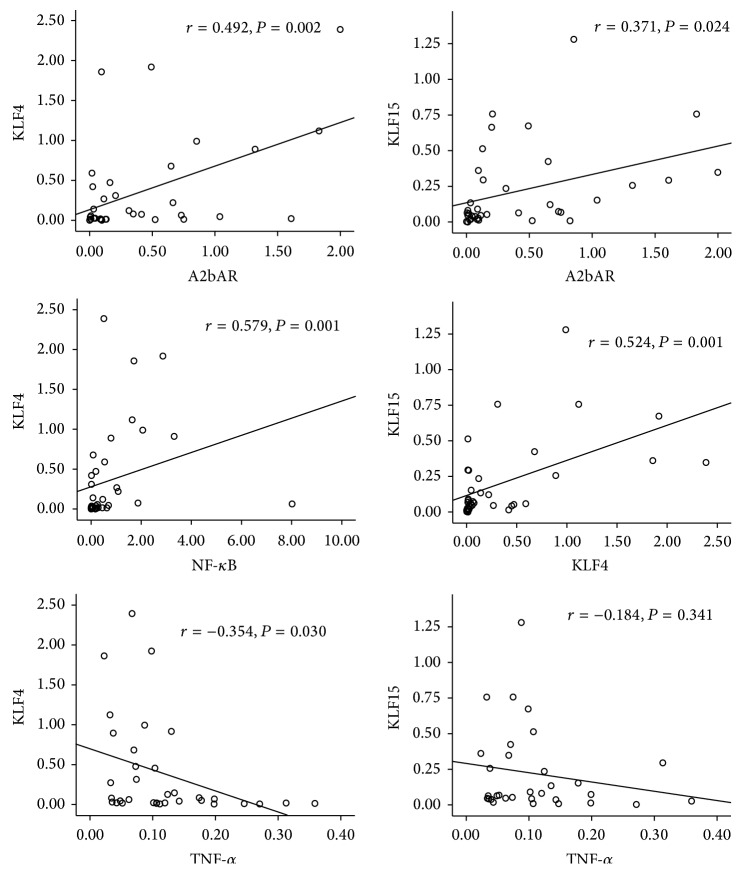
The correlation of* KLF4*,* KLF15,* and the key genes of inflammation signal pathway. Pearson analysis, *P* < 0.05. The correlation between the two groups has statistical significance.

The results were shown in Figure 3. In OB group, the mRNA expression level of* KLF4* was significantly positively correlated with* A2bAR* and* NF-κB* while negatively correlated with* TNF-α* (*P* < 0.05). The mRNA expression level of* KLF15* was significantly positively correlated with* A2bAR* and* KLF4 *(*P* < 0.05) while negatively correlated with* TNF-α*.

## 4. Discussion

Abnormal lipid metabolism induced by obesity is considered one of the core indicators of metabolic syndrome and may be associated with hypertension, dyslipidemia, and T2DM [13]. In the current study, we evaluated the detection rate of dyslipidemia. Our results noted that, in Uygur population, the detection rate of total dyslipidemia was 61.05%, high TC was 14.53%, high TG was 22.67%, low HDL-C was 42.44%, and high LDL-C was 3.49%. More importantly, the detection rates of total dyslipidemia, high TC, high TG, and low HDL-C were higher in Uygur than in Han and Kazak population, which indicates that the Uygur population has more lipid metabolism disorders. The mRNA expression profile chip analysis by our previous research has demonstrated that mRNA expression levels of* KLF4* and* KLF15* were significantly decreased in subjects with obesity, suggesting these genes may play an important role in lipid metabolism in Uygur population.

Recently, it was reported that KLF4-deficient macrophages exhibited lower ability to perform fatty acid oxidation [6]. Moreover, overexpression of KLF4 can increase the M2 macrophages (anti-inflammation) marker protein expression while it can decrease M1 macrophages (inflammation) marker protein expression [6]. KLF4 overexpression reduced the expression of MCP-1 in J774a cells [6]. Recent studies have suggested that KLF15 regulated lipid uptake and utilization in skeletal muscle [14]. In cultured 3T3-L1 adipocytes, treatment with TNF-*α* significantly reduced the mRNA expression of* KLF15* [15]. Moreover,* KLF15* gene ablation attenuated anti-inflammation adipolin expression in adipocytes and KLF15 can significantly attenuate the p300-dependent p65 activation on both the MCP-1 and VCAM-1 promoter [15].

Our findings in Uygur population supported previous chip results and demonstrated that* KLF4* and* KLF15* in the NC group were significantly higher than in the OB group. More importantly, in the OB group, the mRNA expression level of* KLF4* was significantly negatively correlated with BMI, TG, and* TNF-α*. The mRNA expression level of* KLF15* was significantly negatively correlated with TG and LDL and positively correlated with* KLF4*. The above results suggest that the* KLF4* and* KLF15* may collaborate to impact lipid metabolism and inflammation. Interestingly, our results demonstrated that* KLF4* significantly correlated with* NF-κB*. Recent research has found that KLF4 may physically interact with the subunit P65 of NF-*κ*B to limit inflammation in vascular endothelial cells [16]. This research may explain why KLF4 was positively correlated with NF-*κ*B in our study. However, this phenomenon in the adipose tissue has not been reported in the literature to date.

Eisenstein et al. proposed that KLF4 and A2bAR were significantly positively correlated in adipose tissue of American population [17]. A2bAR knockout animals demonstrated elevated liver TG concentrations, which indicated impaired lipid metabolism. Moreover, in A2bAR knockout animals, CCL2, TNF-*α*, and IL-6 level were elevated, whereas IL-10 and IFN-*γ* concentrations were decreased in the epididymal tissue [18]. A2bAR activation ameliorates the course of diabetes and inflammation in low-dose streptozotocin-treated and nonobese diabetic mice [18]. We noted that A2bAR expression level was higher in the NC group as compared to the subjects in the OB group in visceral adipose tissue of Uygur population. Moreover,* A2bAR* significantly correlated with* KLF4* and* KLF15*. The above results indicate that, in the context of obesity, the positive correlation between* A2bAR* and* KLF4/KLF15* may play an important role in obesity-dyslipidemia induced inflammation of visceral adipose tissue in Uygur population.

It is found that free fatty acid can serve as an agonist of the toll-like receptor 4 (TLR4) complex. Stimulation of TLR4 activates proinflammation pathway and induces cytokine expression in various cells [19]. Thus, we evaluated the* TLR4* mRNA expression level. The expression of* TLR4* was significantly higher in the OB group in Uygur population, which indicates that the high level of blood lipid may promote the release of inflammation factors by upregulating the expression of* TLR4*.

Above all, our findings suggest that, in obese status, the lower expression level of* A2bAR*,* KLF4,* and* KLF15* of visceral adipose tissue may correlate with obesity-dyslipidemia induced inflammation in Uygur population.

## Supplementary Material

Supplementary material shows the detection rate of total dyslipidemia, high TC, high TG, low HDL-C and high LDL-C in Han, Uygur and Kazak population. Figure S1and Table S7 showed the chip analysis of differential expression gene in different ethnic group.

## Figures and Tables

**Table 1:** Primers used in real-time PCR of target gene.

Gene	Sequence ID	Primer name	Primer (5′-3′)	Fragment (bp)
*A2bAR*	*XM_011523660.1*	*A2bAR*-F	GGTCATTGCTGTCCTCTG	121
		*A2bAR*-R	TTCATTCGTGGTTCCATCC	
*MYD88*	*XM_006713170.1*	*MYD88*-F	CCGCCTGTCTCTGTTCTTG	115
		*MYD88*-R	GTCCGCTTGTGTCTCCAGT	
*SRC*	*XM_011529014.1*	*MCP-1*-F	CGAGAAAGTGAGACCACGAA	131
		*MCP-1*-R	GTGCGGGAGGTGATGTAGA	
*NF-κB*	*XM_011532009.1*	*NF-κB*-F	CTGAGTCCTGCTCCTTCCA	103
		*NF-κB*-R	CTTCGGTGTAGCCCATTTGT	
*KLF4*	*NM_001314052.1*	*KLF4*-F	GGCACTACCGTAAACACACG	140
		*KLF4*-R	CTGGCAGTGTGGGTCATATC	
*TNF-α*	*NM_000594.3*	*TNF-α*-F	GTGACAAGCCTGTAGCCCAT	111
		*TNF-α*-R	TATCTCTCAGCTCCACGCCA	
*APN*	*NM_004797.3*	*APN*-F	ATGGCCCCTGCACTACTCTA	104
		*APN*-R	CAGGGATGAGTTCGGCACTT	
*MCP-1*	*NM_002982.3*	*MCP-1*-F	GATCTCAGTGCAGAGGCTCG	155
		*MCP-1*-R	TTTGCTTGTCCAGGTGGTCC	
*GAPDH*	*NM_001256799.1*	*GAPDH*-F	GGTGGTCTCCTCTGACTTCAA	211
		*GAPDH*-R	TCTTCCTCTTGTGCTCTTGCT	

**Table 2:** Comparison of subject metrics and biochemical parameters between NC and OB group.

Testing index	NC	OB
Case number	50	45
Age	47.42 ± 17.39	45.94 ± 10.01
Weight (kg)	63.08 ± 7.74	79.96 ± 11.10^*∗∗*^
WC (cm)	89.62 ± 15.29	112.42 ± 8.56^*∗∗*^
HC (cm)	93.40 ± 8.64	106.23 ± 18.23^*∗∗*^
WHR	0.96 ± 0.16	1.07 ± 0.08^*∗∗*^
BMI	22.92 ± 2.54	31.56 ± 3.13^*∗∗*^
SBP (mmHg)	120.52 ± 22.61	129.74 ± 20.95
DBP (mmHg)	80.28 ± 15.2	82.82 ± 14.17
FPG (mmol/L)	5.0 ± 0.85	5.0 ± 0.72
TG (mmol/L)	2.60 ± 1.43	3.76 ± 1.89^*∗∗*^
TC (mmol/L)	4.87 ± 1.20	5.08 ± 1.03
LDL (mmol/L)	2.64 ± 0.80	3.02 ± 0.77^*∗*^
HDL (mmol/L)	1.22 ± 0.47	1.45 ± 0.80

WC: waist circumference; HC: hip circumference; WHR: waist-to-hip ratio; BMI: body mass index; SBP: systolic blood pressure; DBP: diastolic blood pressure; FPG: fasting plasma glucose; TG: triglycerides; TC: cholesterol; HDL: high density lipoproteins; LDL: low density lipoproteins. *t*-test: values are given as the mean ± SD. ^*∗*^ *P* < 0.05, ^*∗∗*^ *P* < 0.01 compared with NC group.
