# Supplementary material for: Correlation of A2bAR and KLF4/KLF15 with Obesity-Dyslipidemia Induced Inflammation in Uygur Population
Source: Mediators Inflamm. 2016 Apr 21;2016:7015620. doi: 10.1155/2016/7015620 (PMC4856914; doi:10.1155/2016/7015620)
Supplement: Supplementary file 1 — Supplementary material shows the detection rate of total dyslipidemia, high TC, high TG, low HDL-C and high LDL-C in Han, Uygur and Kazak population. Figure S1and Table S7 showed the chip analysis of differential expression gene in different ethnic group. [file 7015620.f1.pdf]

The detection rate of total dyslipidemia was 61.05%, and showed a trend of increasing with age; The detection rate of high blood TC was 14.53%, and showed a trend of increasing with age; The detection rate of high blood TG was 22.67%, of which detection rate was decreasing with the age in male, while the detection rate revealed an increasing trend in female ;The detection rate of low HDL-C was 42.44%; The detection rate of high LDL-C was 3.49%.

Table S1 The comparison of the main indexes among the ethnic groups

Detection rate	Age	Male(n,%)	Female(n,%)	Tota;(n,%)
Total dyslipidemia	20-44	50.98(26/51)	58.82(30/51)	54.90(56/102)
	45-59	62.96(17/27)	66.67(14/21)	64.58(31/48)
	60-90	80.00(12/15)	85.71(6/7)	81.82(18/22)
	20-90	59.14(55/93)	63.29(50/79)	61.05(105/172)
High TC	20-44	7.84(4/51)	13.73(7/51)	10.78(11/102)
	45-59	7.40(2/27)	23.81(5/21)	14.58(7/48)
	60-90	26.67(4/15)	42.86(3/7)	31.82(7/22)
	20-90	10.75(10/93)	18.99(15/79)	14.53(25/172)
High TG	20-44	27.45(14/51)	15.69(8/51)	21.57(22/102)
	45-59	25.93(7/27)	19.05(4/21)	22.92(11/48)
	60-90	13.33(2/15)	57.14(4/7)	27.27(6/22)
	20-90	24.73(23/93)	20.25(16/79)	22.67(39/172)
Low HDL-C	20-44	33.33(17/51)	47.06(24/51)	40.20(41/102)
	45-59	48.14(13/27)	42.86(9/21)	45.83(22/48)
	60-90	46.67(7/15)	42.85(3/7)	45.45(10/22)
	20-90	39.78(37/93)	45.56(36/79)	42.44(73/172)
High LDL-C	20-44	3.92(2/51)	3.92(2/51)	3.92(4/102)
	45-59	3.70(1/27)	0(0/21)	2.08(1/48)
	60-90	0(0/15)	1.43(1/7)	4.55(1/22)
	20-90	3.23(3/93)	3.80(3/79)	3.49(6/172)

TG: Triglycerides; TC: Cholesterol; HDL: High density lipoproteins; LDL: Low density lipoproteins. Han (n=600); Uygur (n=172); Kazak (n=630). Comparison between groups was used *t* test. Values are given as the mean ± SD. P<0.01, the difference was statistically significant.

The detection rate of total dyslipidemia in Han, Uygur and Kazak population was respectively 22.22%, 61.05% and 15.08%. The detection rate of total dyslipidemia was significantly higher in Uygur population than Han and Kazak population (P<0.01). The results were shown in the Table S2.

Table S2 The detection rate of dyslipidemia in Han, Uygur and Kazak population

**Ethnics**	**Age**	**Male(n,%)**	**Female(n,%)**	**Total(n,%)**
Han	20-44	32.05(83/259)	9.03(43/476)	17.14(126/735)
	45-59	38.99(62/159)	22.03(65/295)	27.97(127/454)
	60-90	27.66(26/94)	30.26(23/76)	28.82(49/170)
	20-90	33.40(171/512) *#	15.47(131/847) *#	22.22(302/1359) #
Uygur	20-44	50.98(26/51)	58.82(30/51)	54.90(56/102)
	45-59	62.96(17/27)	66.67(14/21)	64.58(31/48)
	60-90	80.00(12/15)	85.71(6/7)	81.82(18/22)
	20-90	59.14(55/93) #	63.29(50/79) #	61.05(105/172) #
Kazak	20-44	13.21(14/106)	6.91(17/246)	8.81(31/352)
	45-59	26.09(18/69)	21.93(25/114)	23.50(43/183)
	60-90	19.51(8/41)	24.07(13/54)	22.11(21/95)
	20-90	18.52(40/216) #	13.29(55/414) #	15.08(95/630) #
Han, Uygur	20-44	29.57(123/416)	11.64(90/773)	17.91(213/1189)
and Kazak	45-59	38.04(97/255)	24.19(104/430)	29.34(201/685)
	60-90	30.67(46/150)	30.66(42/137)	30.66(88/287)
	20-90	32.40(266/821) *	17.61(236/1340) *	23.23(502/2161)

* P<0.01, intra-group comparison; #P<0.01, Comparison among various ethnic groups. The difference was statistically significant.

The detection rate of high TC in Han, Uygur and Kazak population was respectively 6.03%, 14.53% and 11.90%. The detection rate of high TC was significantly higher in Uygur population than Han and Kazak population (P<0.01). The results were shown in the Table S3.

Table S3 The detection rate of high TC in Han, Uygur and Kazak population

**Ethnics**	**Age**	**Male(n,%)**	**Female(n,%)**	**Total(n,%)**
Han	20-44	4.63(12/259)	2.52(12/476)	3.27(24/735)
	45-59	10.69(17/159)	7.80(23/295)	8.81(40/454)
	60-90	6.38(6/94)	15.78(12/76)	10.59(18/170)
	20-90	6.83(35/512) #	5.55(47/847) #	6.03(82/1359) #
Uygur	20-44	7.84(4/51)	13.73(7/51)	10.78(11/102)
	45-59	7.40(2/27)	23.81(5/21)	14.58(7/48)
	60-90	26.67(4/15)	42.86(3/7)	31.82(7/22)
	20-90	10.75(10/93) #	18.99(15/79) #	14.53(25/172) #
Kazak	20-44	8.49(9/106)	3.66(9/246)	5.11(18/352)
	45-59	23.19(16/69)	20.18(23/114)	21.31(39/183)
	60-90	19.51(8/41)	18.52(10/54)	18.95(18/95)
	20-90	15.28(33/216) #	10.15(42/414) #	11.90(75/630) #
Han, Uygur	20-44	6.01(25/416)	3.62(28/773)	4.46(53/1189)
and Kazak	45-59	13.73(35/255)	11.86(51/430)	12.55(86/685)
	60-90	12.0(18/150)	18.25(25/137)	14.98(43/287)
	20-90	9.50(78/821)	7.76(104/1340)	8.42(182/2161)

#P<0.01, Comparison among various ethnic groups. The difference was statistically significant.

The detection rate of high TG in Han, Uygur and Kazak population was respectively 12.36%, 22.67% and 3.02%. The detection rate of high TG was significantly higher in Uygur population than Han and Kazak population (P<0.01). The results were shown in the Table S4.

Table S4 The detection rate of high TG in Han, Uygur and Kazak population

Ethnics	Age	Male(n,%)	Female(n,%)	Total(n,%)
Han	20-44	16.60(43/259)	3.99(19/476)	8.44(62/735)
	45-59	25.16(40/159)	12.88(38/295)	17.18(78/454)
	60-90	15.96(15/94)	17.11(13/76)	16.47(28/170)
	20-90	19.14(98/512) *#	8.26(70/847) *#	12.36(168/1359) #
Uygur	20-44	27.45(14/51)	15.69(8/51)	21.57(22/102)
	45-59	25.93(7/27)	19.05(4/21)	22.92(11/48)
	60-90	13.33(2/15)	57.14(4/7)	27.27(6/22)
	20-90	24.73(23/93) #	20.25(16/79) #	22.67(39/172) #
Kazak	20-44	6.60(7/106)	1.63(4/246)	3.13(11/352)
	45-59	4.35(3/69)	1.75(2/114)	2.73(5/183)
	60-90	0(0/41)	5.56(3/54)	3.16(3/95)
	20-90	4.63(10/216) #	2.17(9/414) #	3.02(19/630) #
Han, Uygur	20-44	15.38(64/416)	4.01(31/773)	7.99(95/1189)
and Kazak	45-59	19.61(50/255)	10.23(44/430)	13.72(94/685)
	60-90	11.33(17/150)	14.60(20/137)	12.89(37/287)
	20-90	15.96(131/821) *	7.10(95/1340) *	10.46(226/2161)

* P<0.01, intra-group comparison; #P<0.01, Comparison among various ethnic groups. The difference was statistically significant.

The detection rate of low HDL-C in Han, Uygur and Kazak population was respectively 6.11%, 42.44% and 0.63%. The detection rate of low HDL-C was significantly higher in Uygur population than Han and Kazak population ($P<0.01$). The results were shown in the Table S5.

Table S5 The detection rate of low HDL-C in Han,Uygur and Kazak population

Ethnics	Age	Male(n,%)	Female(n,%)	Total(n,%)
Han	20-44	13.13(34/259)	9.66(16/476)	6.80(50/735)
	45-59	10.06(16/159)	2.37(7/295)	5.07(23/454)
	60-90	7.45(7/94)	3.95(3/76)	5.88(10/170)
	20-90	11.13(57/512) *#	3.07(26/847) *#	6.11(83/1359) #
Uygur	20-44	33.33(17/51)	47.06(24/51)	40.20(41/102)
	45-59	48.14(13/27)	42.86(9/21)	45.83(22/48)
	60-90	46.67(7/15)	42.85(3/7)	45.45(10/22)
	20-90	39.78(37/93) #	45.56(36/79) #	42.44(73/172) #
Kazak	20-44	0(0/109)	1.63(4/246)	1.14(4/352)
	45-59	0(0/69)	0(0/114)	0(0/183)
	60-90	0(0/41)	0(0/54)	0(0/95)
	20-90	0(0/216) #	0.97(4/414) #	0.63(4/630) #
Han, Uygur	20-44	12.26(51/416)	5.69(44/773)	7.99(95/1189)
and Kazak	45-59	11.37(29/255)	3.72(16/430)	6.57(45/685)
	60-90	9.33(14/150)	4.38(6/137)	6.97(20/287)
	20-90	11.45(94/821) *	4.93(66/1340) *	7.40(160/2161)

* $P<0.01$, intra-group comparison; #$P<0.01$, Comparison among various ethnic groups. The difference was statistically significant.

The detection rate of high LDL-C in Han, Uygur and Kazak population was respectively 5.30%, 3.49% and 5.71%, and the difference was not significant. The results were shown in the Table S6.

Table S6 The detection rate of high LDL-C in Han,Uygur and Kazak participants

Ethnics	Age	Male(n,%)	Female(n,%)	Total(n,%)
Han	20-44	6.17(16/259)	1.26(6/476)	2.99(22/735)
	45-59	13.21(21/159)	6.10(18/295)	8.59(39/454)
	60-90	3.19(3/94)	10.53(8/76)	6.47(11/170)
	20-90	7.81(40/512) *	3.78(32/847) *	5.30(72/1359)
Uygur	20-44	3.92(2/51)	3.92(2/51)	3.92(4/102)
	45-59	3.70(1/27)	0(0/21)	2.08(1/48)
	60-90	0(0/15)	1.43(1/7)	4.55(1/22)
	20-90	3.23(3/93)	3.80(3/79)	3.49(6/172)
Kazak	20-44	1.89(2/106)	2.03(5/246)	1.99(7/352)
	45-59	13.04(9/69)	8.77(10/114)	10.38(19/183)
	60-90	9.76(4/41)	11.11(6/54)	10.53(10/95)
	20-90	6.94(15/216)	5.07(21/414)	5.71(36/630)
Han, Uygur and Kazak	20-44	4.81(20/416)	1.68(13/773)	2.76(33/1189)
	45-59	12.16(31/255)	6.51(28/430)	8.61(59/685)
	60-90	4.67(7/150)	10.95(15/137)	7.67(22/287)
	20-90	7.06(58/821) *	4.18(56/1340) *	5.28(114/2161)

* $P<0.01$, intra-group comparison; #$P<0.01$, Comparison among various ethnic groups. The difference was statistically significant.

In the Han ethnic group, the microarray analysis results indicated that compared with the normal group, the obesity group had 3 genes (i.e., *ITGB1*, *ITGA7*,*CCND1*) that were significantly up expressed, and 3 genes (i.e., *MYC5*,*CXCR34*,*ALDH1A23*) that were significantly down expressed. Within the Uygur ethnic group, the obesity group had 10 genes (i.e., ***KLF4*,*KLF15*,***PIK3CD*, *MYD88*, *RAC2*, *JAK3*, *CCR1*, *CXCR2*, *PPKCB*, *VCAM1*) that were significantly down expressed. Within the Kazak ethnic group, the obesity group had 2 genes (i.e., *PLA2G2D*, *UGT2A1*) that were significantly under expressed. The results were shown in the Figure S1 and Table S1.

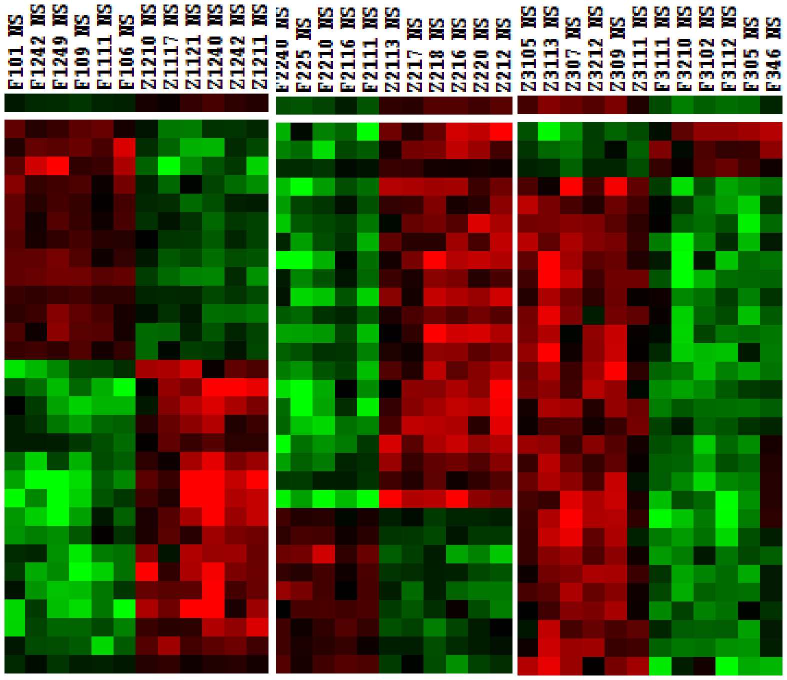

A B C

Figure S1 Differential expression gene in different ethnic group.

A. Han ethnic group; B. Uygur ethnic group; C. Kazak ethnic group

Table S7 Differentially expressed genes among the Han, Uygur and Kazaks ethnic group

	**Han**		**Uygur**		**Kazak**	
	**Up**	**Down**	**Up**	**Down**	**Up**	**Down**
**Obesity VS Normal**	*ITGB1(9), ITGA7(8), CCND1(4)*	*MYC(5),CXCR3(4), ALDH1A2(3)*	/	***KLF4(10), KLF15(9),*** *PIK3CD(10), MYD88(8), RAC2(7), JAK3(7), CCR1(7), CXCR2(7), PPKCB(7), VCAM1(6)*	/	*PLA2G2D(4), UGT2A1(3)*

The brackets indicate the fold change (in multiples) of the differently expressed genes.

The weight, BMI, WC, HC, WHR, Fasting blood-glucose, Fasting Insulin, TG and TC in the Uygur population were significantly higher than those in the Han and Kazak groups (*P*<0.01). The HDL in the Uygur population was significantly lower than that in the Han and Kazak (*P*<0.01). The LDL in the Uygur population was significantly lower than Han, while higher than Kazak (*P*<0.01). The results were shown in the Table S7.

Table S8 The comparison of the main indexes among the ethnic groups

Indexes	Ethnics	n	Mean	SD	95% CI		F	P
					Lower	Upper		
**Height**	**Han**	600	162.58	7.49	161.98	163.18	4.011	0.018
**(cm)**	**Uygur**	172	160.49	8.28	159.24	161.74		
	**Kazak**	630	161.70	10.32	160.89	162.51		
**Weight**	**Han**	600	64.06	11.00	63.18	64.94	102.318	0.000
**(kg)**	**Uygur**	172	79.39	9.73	77.92	80.85		
	**Kazak**	630	68.24	14.23	67.13	69.35		
**BMI**	**Han**	600	24.14	3.13	23.89	24.39	198.255	0.000
**(Kg/M^2)**	**Uygur**	172	30.77	2.59	30.38	31.16		
	**Kazak**	630	25.95	4.70	25.58	26.32		
**WC**	**Han**	600	81.65	9.92	80.85	82.44	166.415	0.000
**(cm)**	**Uygur**	172	99.43	8.35	98.18	100.69		
	**Kazak**	630	84.67	13.12	83.64	85.69		
**HC**	**Han**	600	94.99	6.51	94.46	95.51	166.793	0.000
**(cm)**	**Uygur**	172	107.59	7.07	106.53	108.66		
	**Kazak**	630	98.91	9.52	98.17	99.66		
**WHR**	**Han**	600	0.86	0.09	0.85	0.87	50.204	0.000
	**Uygur**	171	0.92	0.06	0.92	0.93		
	**Kazakh**	630	0.85	0.08	0.85	0.86		
**SBP**	**Han**	600	120.26	15.56	119.01	121.50	22.550	0.000
**(mmHg)**	**Uygur**	165	120.58	20.64	117.41	123.75		
	**Kazak**	630	127.50	23.02	125.69	129.30		

**DBP**	**Han**	600	74.81	10.78	73.94	75.67	91.297	0.000
**(mmHg)**	**Uygur**	165	78.81	13.17	76.78	80.83		
	**Kazak**	630	84.77	14.70	83.62	85.92		
**Fasting**	**Han**	600	5.79	1.36	5.68	5.90	121.381	0.000
**blood-glucose**	**Uygur**	172	6.05	1.09	5.89	6.21		
**(mmol/L)**	**Kazak**	630	4.84	1.11	4.75	4.92		
**2 hour**	**Han**	600	7.74	3.84	7.43	8.05	62.599	0.000
**blood-glucose**	**Uygur**	172	6.58	2.33	6.23	6.93		
**(mmol/L)**	**Kazak**	630	5.81	2.23	5.63	5.98		
**Fasting Insulin**	**Han**	599	10.52	10.36	9.69	11.36	16.890	0.000
**(mmol/L)**	**Uygur**	164	13.41	23.54	9.78	17.04		
	**Kazak**	630	7.81	8.67	7.14	8.49		
**2 hour**	**Han**	600	52.17	50.60	48.12	56.23	107.390	0.000
**Insulin**	**Uygur**	171	33.31	39.15	27.40	39.22		
**(mmol/L)**	**Kazak**	630	19.64	22.84	17.85	21.42		
**TC**	**Han**	600	5.00	0.96	4.92	5.08	5.429	0.004
**(mmol/L)**	**Uygur**	172	5.26	1.06	5.10	5.42		
	**Kazak**	630	4.98	1.04	4.90	5.06		
**TG**	**Han**	600	1.64	1.99	1.48	1.80	107.952	0.000
**(mmol/L)**	**Uygur**	172	2.47	1.30	2.28	2.67		
	**Kazak**	630	0.83	0.57	0.78	0.87		
**HDL**	**Han**	600	1.39	0.30	1.37	1.42	292.661	0.000
**(mmol/L)**	**Uygur**	172	1.18	0.34	1.13	1.23		
	**Kazak**	630	1.67	0.21	1.65	1.69		
**LDL**	**Han**	600	3.22	0.75	3.16	3.28	30.178	0.000
**(mmol/L)**	**Uygur**	172	3.01	0.51	2.93	3.08		
	**Kazak**	630	2.90	0.74	2.84	2.96		

WC: waist circumference ;HC: hip circumference; WHR: waist-to-hip ratio ;BMI : Body mass index; SBP: Systolic blood pressure; DBP: Diastolic blood pressure; HDL: High density lipoproteins; TG: Triglycerides; TC: Cholesterol; LDL: Low density lipoproteins. Han (n=600); Uygur (n=172); Kazak

(n=630). Comparison between groups was used One-way analysis of variance (ANOVA). Values are given as the mean ± SD. $P<0.01$, the difference was statistically significant.

The levels of serum lipid (TC, TG, HDL, and LDL) were significantly correlated with height, weight, BMI, WC, HC, WHR, SBP, DBP, fasting plasma glucose, 2-hour plasma glucose, fasting insulin, and 2-hour insulin in Han and Kazak populations; while, interestingly, the correlations of serum lipid levels and other indices only reflected two relationships in the Uygur population: TC was positively correlated with height, and LDL was positively correlated with 2-hour plasma glucose. These results suggested that the high lipid levels might be mainly related to genetic factors in the Uygur population. The results were shown in the Table S8.

Table S9 The Correlation of the main indexes

Ethnics	Indexes	Correlation	Height	Weight	BMI	WC	HC	WHR	SBP	DBP	Fasting blood glucose	2 hour plasma glucose	Fasting Insulin	2 hour Insulin	TC	TG	HDL	LDL
**Han**	**Height**	r	1.000	0.646	0.172	0.350	0.221	0.298	0.111	0.205	0.096	0.095	0.025	0.023	0.018	0.147	-0.193	0.112
		p	.	***0.000***	***0.000***	***0.000***	***0.000***	***0.000***	***0.006***	***0.000***	***0.019***	***0.020***	0.539	0.566	0.665	***0.000***	***0.000***	***0.006***
	**Weight**	r		1.000	0.845	0.756	0.618	0.551	0.354	0.403	0.231	0.255	0.245	0.245	0.135	0.395	-0.346	0.350
		p		.	***0.000***	***0.000***	***0.000***	***0.000***	***0.000***	***0.000***	***0.000***	***0.000***	***0.000***	***0.000***	***0.001***	***0.000***	***0.000***	***0.000***
	**BMI**	r			1.000	0.742	0.669	0.502	0.391	0.389	0.231	0.264	0.321	0.308	0.173	0.421	-0.313	0.392
		p			.	***0.000***	***0.000***	***0.000***	***0.000***	***0.000***	***0.000***	***0.000***	***0.000***	***0.000***	***0.000***	***0.000***	***0.000***	***0.000***
	**WC(cm)**	r				1.000	0.582	0.851	0.436	0.411	0.350	0.372	0.283	0.240	0.201	0.450	-0.325	0.400
		p					***0.000***	***0.000***	***0.000***	***0.000***	***0.000***	***0.000***	***0.000***	***0.000***	***0.000***	***0.000***	***0.000***	***0.000***
	**HC(cm)**	r					1.000	0.115	0.254	0.270	0.199	0.126	0.282	0.142	0.066	0.233	-0.163	0.200
		p					.	***0.005***	***0.000***	***0.000***	***0.000***	***0.002***	***0.000***	***0.001***	***0.109***	***0.000***	***0.000***	***0.000***
	**WHR**	r						1.000	0.384	0.349	0.309	0.390	0.172	0.213	0.209	0.412	-0.294	0.367
		p						.	***0.000***	***0.000***	***0.000***	***0.000***	***0.000***	***0.000***	***0.000***	***0.000***	***0.000***	***0.000***
	**SBP**	r							1.000	0.635	0.249	0.394	0.182	0.197	0.223	0.333	-0.136	0.319
		p							.	***0.000***	***0.000***	***0.000***	***0.000***	***0.000***	***0.000***	***0.000***	***0.001***	***0.000***
	**DBP**	r								1.000	0.247	0.315	0.183	0.232	0.197	0.335	-0.150	0.293
		p								.	***0.000***	***0.000***	***0.000***	***0.000***	***0.000***	***0.000***	***0.000***	***0.000***
	**Fasting plasma**	r									1.000	0.559	0.293	0.137	0.177	0.312	-0.157	0.302
	**glucose**	p									.	***0.000***	***0.000***	***0.001***	***0.000***	***0.000***	***0.000***	***0.000***
	**2 hour plasma**	r										1.000	0.178	0.411	0.226	0.396	-0.196	0.363
	**glucose**	p										.	***0.000***	***0.000***	***0.000***	***0.000***	***0.000***	***0.000***
	**Fasting Insulin**	r											1.000	0.360	0.174	0.296	-0.197	0.293
		p												***0.000***	***0.000***	***0.000***	***0.000***	***0.000***
	**2 hour**	r												1.000	0.143	0.334	-0.204	0.285

	**Insulin**	p												.	***0.000***	***0.000***	***0.000***	***0.000***
	**TC**	r													1.000	0.430	0.229	0.739
		p													.	***0.000***	***0.000***	***0.000***
	**TG**	r														1.000	-0.319	0.771
		p														.	***0.000***	***0.000***
	**HDL**	r															1.000	-0.220
		p															.	***0.000***
**Uygur**	**Height**	r	1.000	0.739	-0.123	0.304	0.159	0.252	0.107	0.063	0.101	-0.013	0.156	0.025	-0.177	0.030	-0.042	-0.052
		p	.	***0.000***	0.108	***0.000***	***0.037***	***0.001***	0.171	0.421	0.188	0.865	***0.047***	0.749	***0.020***	0.698	0.589	0.501
	**Weight**	r		1.000	0.495	0.575	0.428	0.317	0.209	0.184	0.168	0.061	0.239	0.009	-0.094	0.057	0.002	-0.044
		p		.	***0.000***	***0.000***	***0.000***	***0.000***	***0.007***	***0.018***	***0.028***	0.424	***0.002***	0.911	0.220	0.461	0.978	0.567
	**BMI**	r			1.000	0.437	0.422	0.147	0.186	0.197	0.098	0.118	0.234	-0.031	0.053	0.131	0.003	0.051
		p			.	***0.000***	***0.000***	0.055	***0.017***	***0.011***	0.199	0.123	***0.003***	0.686	0.494	0.086	0.965	0.508
	**WC**	r				1.000	0.665	0.591	0.126	0.172	0.165	0.074	0.237	0.039	0.128	0.117	0.065	-0.008
		p				.	***0.000***	***0.000***	0.108	***0.027***	***0.031***	0.336	***0.002***	0.612	0.095	0.127	0.400	0.917
	**HC**	r					1.000	-0.137	0.010	0.090	0.117	0.166	0.198	0.073	0.040	0.065	0.067	0.071
		p					.	0.075	0.898	0.253	0.125	***0.030***	***0.011***	0.346	0.606	0.394	0.382	0.354
	**WHR**	r						1.000	0.170	0.131	0.137	-0.093	0.129	-0.029	0.121	0.150	-0.008	-0.050
		p						.	***0.029***	0.093	0.075	0.227	0.101	0.704	0.114	0.050	0.915	0.519
	**SBP**	r							1.000	0.864	0.115	0.140	0.116	0.107	0.153	0.101	0.142	0.021
		p							.	***0.000***	0.142	0.072	0.144	0.172	0.050	0.195	0.070	0.791
	**DBP**	r								1.000	0.083	0.160	0.049	0.106	0.165	0.069	0.141	-0.040
		p								.	0.289	0.040	0.541	0.175	0.034	0.376	0.070	0.613
	**Fasting plasma**	r									1.000	0.399	0.310	0.199	0.026	0.104	0.083	0.077
	**glucose**	p									.	***0.000***	***0.000***	***0.009***	0.732	0.177	0.282	0.315
	**2 hour plasma**	r										1.000	0.182	0.564	0.087	0.138	0.132	0.196
	**glucose**	p										.	***0.020***	***0.000***	0.256	0.071	0.083	***0.010***
	**Fasting Insulin**	r											1.000	0.267	0.029	0.113	0.096	0.098
		p											.	***0.001***	0.714	0.149	0.224	0.211
	**2 hour**	r												1.000	-0.009	0.103	0.065	0.148
	**Insulin**	p												.	0.905	0.182	0.396	0.054
	**TC**	r													1.000	0.230	0.243	0.221
		p													.	***0.002***	***0.001***	***0.004***
	**TG**	r														1.000	0.030	0.270

		p															0.699	***0.000***
	HDL	r															1.000	0.040
		p															.	0.605
Kazak	Height	r	1.000	0.440	-0.024	0.101	0.081	0.098	-0.026	-0.051	0.018	-0.085	0.064	-0.126	-0.021	0.091	0.023	-0.053
		p	.	***0.000***	0.551	***0.011***	***0.042***	***0.014***	0.513	0.202	0.648	***0.032***	0.108	***0.002***	0.599	***0.022***	0.568	0.182
	Weight	r		1.000	0.869	0.767	0.792	0.492	0.283	0.305	0.083	0.185	0.232	0.146	0.214	0.431	0.220	0.136
		p		.	***0.000***	***0.000***	***0.000***	***0.000***	***0.000***	***0.000***	***0.038***	***0.000***	***0.000***	***0.000***	***0.000***	***0.000***	***0.000***	***0.001***
	BMI	r			1.000	0.811	0.840	0.516	0.338	0.378	0.085	0.242	0.230	0.227	0.270	0.437	0.242	0.194
		p			.	***0.000***	***0.000***	***0.000***	***0.000***	***0.000***	***0.034***	***0.000***	***0.000***	***0.000***	***0.000***	***0.000***	***0.000***	***0.000***
	WC	r				1.000	0.829	0.823	0.387	0.402	0.080	0.267	0.148	0.222	0.316	0.462	0.334	0.244
		p				.	***0.000***	***0.000***	***0.000***	***0.000***	***0.044***	***0.000***	***0.000***	***0.000***	***0.000***	***0.000***	***0.000***	***0.000***
	HC	r					1.000	0.394	0.275	0.324	0.109	0.240	0.176	0.194	0.252	0.385	0.245	0.205
		p					.	***0.000***	***0.000***	***0.000***	***0.006***	***0.000***	***0.000***	***0.000***	***0.000***	***0.000***	***0.000***	***0.000***
	WHR	r						1.000	0.371	0.347	0.020	0.202	0.063	0.157	0.279	0.391	0.304	0.199
		p						.	***0.000***	***0.000***	0.616	***0.000***	0.116	***0.000***	***0.000***	***0.000***	***0.000***	***0.000***
	SBP	r							1.000	0.773	-0.002	0.237	0.052	0.151	0.325	0.307	0.325	0.255
		p								***0.000***	0.965	***0.000***	0.191	***0.000***	***0.000***	***0.000***	***0.000***	***0.000***
	DBP	r								1.000	0.061	0.192	0.126	0.204	0.311	0.308	0.303	0.255
		p									0.126	***0.000***	***0.002***	***0.000***	***0.000***	***0.000***	***0.000***	***0.000***
	Fasting plasma	r									1.000	0.363	0.121	0.186	0.131	0.149	0.023	0.119
	glucose	p									.	***0.000***	***0.002***	***0.000***	***0.001***	***0.000***	0.568	***0.003***
	2 hour plasma	r										1.000	0.113	0.375	0.197	0.212	0.119	0.159
	glucose	p										.	***0.004***	***0.000***	***0.000***	***0.000***	***0.003***	***0.000***
	Fasting Insulin	r											1.000	0.361	0.109	0.211	0.015	0.063
		p											.	***0.000***	***0.006***	***0.000***	0.710	0.115
	2 hour	r												1.000	0.161	0.216	0.069	0.148
	Insulin	p												.	***0.000***	***0.000***	0.086	***0.000***
	TC	r													1.000	0.411	0.639	0.893
		p													.	***0.000***	***0.000***	***0.000***
	TG	r														1.000	0.327	0.252
		p														.	***0.000***	***0.000***
	HDL	r															1.000	0.584
		p																***0.000***

Spearman analysis, $P < 0.01$, $P < 0.05$.The difference between the two groups has statistical significance.
